# Reese’s Analysis of Physiology

**Published:** 1852-06

**Authors:** 


*An Analysis of Physiology, being a condensed view of its most important facts and doctrines, designed especially for the use of Students.* By JOHN J. REESE, M.D., Lecturer on Materia Medica and Therapeutics in the Medical Institute of Philadelphia; Physician of Wills Hospital; Fellow of the College of Physicians. Second edition revised and enlarged. pp. 368, 12mo. Phil.: Lindsay & Blakiston, 1852. From the publishers, through Messrs. S. C. GRIGGS & Co.

BELIEVING that the old adage is still correct that "there is no royal road to knowledge"—that the student in any science or profession must labor faithfully and diligently before he can acquire sufficient information to allow him honestly and justly to practice his profession, we have had but little respect for "Handbooks," "Manuels," &c., compilations intended as labor-saving works,—making their readers superficial and conservative in their ideas, and as a consequence, reflecting but little honor upon the profession of which they are members.

Entertaining such feelings we were agreeably surprised, upon examining the work to find it far superior to what its title led us to suppose, a manual for the use of students. It is, as it indicates, a condensed view of the most important facts and doctrines of Physiology as believed by the scientific men of our profession at the present day, and is worthy of a place, not only among the text-books of the student, but in the library of the physician, as a book for reference and instruction; and as such, we would therefore recommend it to our medical friends for their examination. B.

